# Selective modification of Ti6Al4V surfaces for biomedical applications†

**DOI:** 10.1039/c9ra11000c

**Published:** 2020-05-06

**Authors:** Gabriela Melo Rodriguez, James Bowen, Mischa Zelzer, Artemis Stamboulis

**Affiliations:** Biomaterials Group, School of Metallurgy and Materials, University of Birmingham Edgbaston Birmingham B15 2TT UK a.stamboulis@bham.ac.uk; School of Engineering and Innovation, The Open University Walton Hall Milton Keynes MK7 6AA UK; School of Pharmacy, University of Nottingham Nottingham NG7 2RD UK

## Abstract

The surface of a medical implant is required to interact favourably with ions, biomolecules and cells *in vivo*, commonly resulting in the formation of the extracellular matrix. Medical grade Ti6Al4V alloy is widely used in orthopaedic and dental applications for bone replacement due to its advantageous mechanical properties and biocompatibility, which enhances the adhesion between native tissue and the implanted material. In this study, chemical and thermal modification of a medical-grade Ti6Al4V alloy were performed to enhance electrostatic interactions at the alloy surface with a synthetic peptide, suitable for conferring drug release capabilities and antimicrobial properties. The modified surfaces exhibited a range of topographies and chemical compositions depending primarily on the treatment temperature. The surface wetting behaviour was found to be pH-dependent, as were the adhesive properties, evidenced by chemical force titration atomic force microscopy.

## Abstract 2

The surface of a medical implant is required to interact favourably with ions, biomolecules and cells *in vivo*, commonly resulting in the formation of the extracellular matrix.

## Graphical abstract

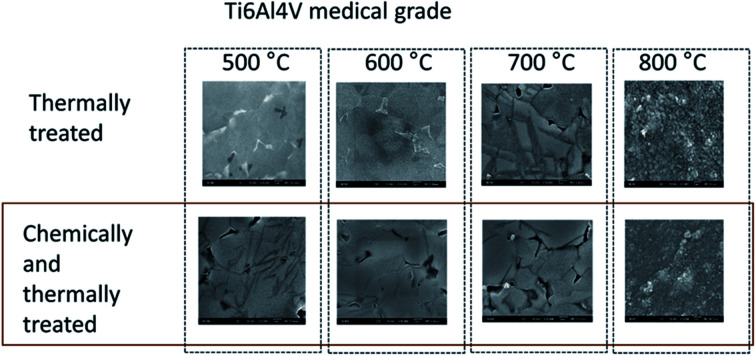

## 1 Introduction

Titanium and its alloys are excellent materials for orthopaedic and dental implants due to their mechanical properties, including those conferred by the surface such as resistance to corrosion and bioactivity.^1^ Titanium surfaces present negative charges when in contact with aqueous solutions at physiological pH (around 7.4) attributed to the native titanium oxide (TiO_*x*_). The composition of the native TiO_*x*_ surface is primarily TiO_2_ in amorphous phase.^2,3^ It was shown that the crystal phases of anatase and rutile present on the surface offer better bioactivity than the amorphous phase^4–6^ and therefore the presence of these phases are more desirable for cell proliferation. A method commonly used for the crystallisation of amorphous TiO_2_ is thermal oxidation. During this method, the formation of crystal phases is highly dependent on the temperature. For example, the TiO_2_ layer formed following thermal oxidation treatment at temperatures below 500 °C is mainly amorphous with some anatase present.^7,8^ After the treatment at a temperature of 550 °C, both rutile and anatase crystal phases are present.^7,9,10^ In contrast, oxidation treatment at temperatures over 800 °C leads to the formation of the rutile phase only.^9,11^

The surface topography and charge density of Ti and its alloys are properties that can be modified by chemical treatments.^7,12–14^ One approach to alter these surfaces chemically is to use H_2_O_2_ in aqueous solution. This modification produces a titania gel layer (TGL) rich in OH^−^ with nano and microporosity^8,15^ which is desirable for the adsorption of ions, peptides, and proteins, as well as the promotion of cell adhesion.^16–18^ Furthermore, the TGL on the surface can protect the supporting metal from corrosion in the biological environment.^7,18^ This is particularly relevant to the use of titanium for implantation. This chemical treatment, combined with a thermal treatment, form a TiO_*x*_ surface that can prevent the liberation of cytotoxic elements from the bulk titanium into the surrounding tissues. This is supported by Browne and Gregson, who found that the release of titanium and aluminium ions from the oxide films on Ti6Al4V annealed at 400 °C for 0.75 h was reduced from 0.14 to 0.03 μg cm^−2^ (Ti), and from 0.25 to 0.026 (Al) μg cm^−2^, respectively, compared with the standard nitric-acid passivated surface.^19^ Control over the chemical moieties, charge density, and topography of the surface can be utilised for a multitude of applications. For example, rough surfaces can lead to the formation and deposition of calcium phosphates. The latter intervenes as anchorage for collagenous fibres produced by bone cells or osteoblasts, and in most of the cases helps to introduce biocompatibility.^20^

A previous study reported that a Ti6Al4V surface treated by H_2_O_2_ exhibited improved electrostatic adsorption of peptides;^21^ however, the surface properties were not reported. This study aims to show how the thermal oxidation and chemical treatment with H_2_O_2_ of a medical-grade Ti6Al4V surface can affect its chemical composition and topography in the view of the surface capability to interact with organic molecules such as peptides for drug delivery applications and antimicrobial properties.

## 2 Experimental

### 2.1 Titanium alloy surface sample preparation prior to surface modifications

Single-sided-polished Ti6Al4V plates (ELI ASTM F136-2002a) were supplied by Titanium Industries UK Ltd. The bulk material composition was reported by the manufacturer and was: wt% in the top O 0.13%, V 4.12%, C 0.02%, Ti >88.854%, N 0.02%, Al 6.42%, H 0.006%, Fe 0.03% and in the bottom O 0.13%, V 4.14%,C 0.02%, Ti 88.864%, N 0.02%, Al 6.38%, H 0.006%, Fe 0.04%. For both sides, each residual elements were <0.10% and the residual elements total were <0.40%. The method of characterization is described in ASTM F136-13 Standard.^22^ The Ti6Al4V plates were cut using a guillotine into square plates of dimensions 10 mm × 10 mm. The 1.6 mm thick plates were then polished until a mirror-like finish following the Struers polishing protocol.^23^ The plates were then washed following 15 min of consequent ultrasonic baths with deionised water (dH_2_O), acetone, and finally, dH_2_O, followed by a drying process in air at room temperature. This is the initial surface procedure used prior to the chemical and thermal modifications and is denoted by MPT.

#### 2.1.1 Chemical treatment

A mixture of 90 v/v % of 9.78 M H_2_O_2_ supplied by Sigma Aldrich (chemical grade), and 10 v/v % of 1 M HCl supplied by Merck Millipore (analytical grade) was prepared. This solution was used to etch the surface of the metallic samples. Cleaned and dried Ti6Al4V samples were reacted with 10 mL of the etching solution at 80°C in an oven (Pickstone, UK) for 30 min. The plates were then rinsed with dH_2_O and dried in air at room temperature.

#### 2.1.2 Thermal oxidation in air

Untreated and chemically treated Ti6Al4V plates were thermally oxidised in an air furnace (Lenton, UK) at 500, 600, 700 and 800 °C. Upon reaching the set point temperature, the samples were further heat-treated for 1 h. The samples were then removed from the oven and left to cool in the air at room temperature. The samples were stored under air in sealed containers. All analyses were performed within 90 days of the modification procedure. Samples description and abbreviations are in Table 1.

**Table 1:** Sample list description and abbreviation

Ti6Al4V sample treatment	Abbreviation
Polished surfaces	MPT
Thermally oxidised	TO
Chemically treated	TGL
Thermally treated	TO_*n*_ (*n* = 500, 600, 700 and/or 800 °C)
Chemically and thermally treated	TGL-TO_*n*_ (*n* = 500, 600, 700 and/or 800 °C)

### 2.2 Characterization

#### 2.2.1 Scanning electron microscopy analysis

SEM micrographs of the untreated and treated surfaces were obtained using a JSM-7000F SEM (JEOL, Japan), operating with an electron voltage of 20 kV.

#### 2.2.2 X-ray diffraction analysis

X-ray diffractograms of untreated and treated surfaces were obtained using a D8 Advance XRD (Bruker, Germany) with Bragg Brentano mode and Cu Kα (*λ* = 1.64 Å) in the 2*θ* between 20–60° using a step size of 0.02°. The obtained data were analysed using Match software (Crystal Impact, Germany).

#### 2.2.3 Raman spectroscopy analysis

Raman spectroscopy of untreated and treated surfaces was performed using a LabRAM HR 800 Raman microscope (Horiba Jobin Yvon, Japan) with a laser source of wavelength 532 nm. The analysed areas were 60 μm × 70 μm, and the data were acquired for 45 s, in three non-overlapping locations. The data were analysed by LabSpec 5 software (Horiba, Japan).

#### 2.2.4 AFM imaging analysis

AFM images of untreated and treated surfaces were obtained using a NanoWizard II AFM (JPK Instruments, UK) operating in intermittent contact mode at 18 °C and relative humidity of <40%. Rectangular pyramidal-tipped Si cantilevers (PPP-NCL, Windsor Scientific, UK) were employed (nominal length and width were 225 μm and 38 μm respectively; their nominal tip diameter was <10 nm). The nominal frequency of the first resonant mode was 190 kHz. A tip velocity of 10 μm s^−1^ was employed when scanning across the sample surface. Each image was composed of 512 × 512 pixels and took approximately 20 min to acquire. Images were analysed using a JPK Data Processing software (JPK Instruments, UK) and a Scanning Probe Image Processor software (Image Metrology, Denmark).

#### 2.2.5 Dynamic contact angle analysis

Dynamic contact angle (DCA) data of all surfaces were obtained using a Theta OneAttension optical tensiometer (Biolin Scientific, Sweden). Advancing contact angle (*θ*_a_) data were obtained in triplicate, on three different samples, with acidic and alkaline solutions at pH 3, 5, 7 and 9. The drop size was 2 μL, and the rate of volume change was 0.5 μL s^−1^. Data were analysed with a Young–Laplace curve fitting, using OneAttension software (Biolin Scientific, Sweden).

#### 2.2.6 AFM adhesion analysis

Acquisition of adhesion data was performed using a NanoWizard II AFM (JPK Instruments, UK) employing a CellHesion module (JPK Instruments, UK), operating in force spectroscopy mode at 18°C. The sample and cantilever were immersed in acidic and alkaline aqueous solutions, contained within a clean glass Petri dish. The solutions were manufactured using dH_2_O and pH adjusted with NaOH 0.01 M or HCl 0.01 M, to achieve solutions of pH 3, 5, 7 and 9. The samples were immobilised using a double-sided Shintron adhesive tape (Agar Scientific, UK).

The vertical deflection and *z*-axis displacement data were recorded at a frequency of 10 kHz. A grid of 100 force-displacement curves was acquired for each sample/liquid combination, equally spaced over an area of 100 μm × 100 μm. The force-displacement data were analysed using JPK Data Processing software (JPK Instruments, UK).

**Fig. 1:**
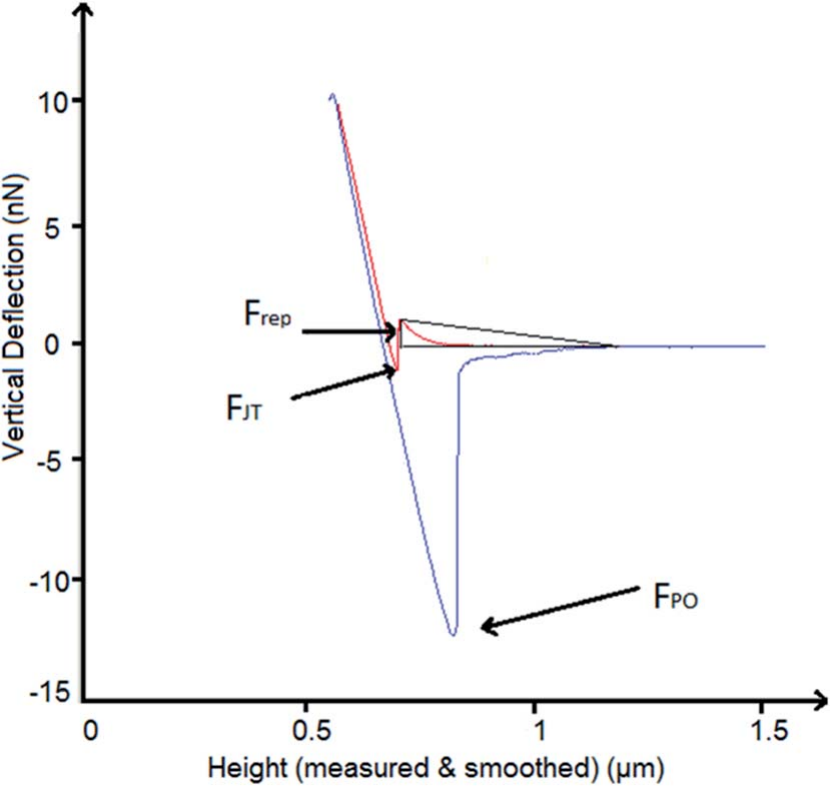
Description of measured forces in the force–distance curves obtained by AFM. In red, the approach curve and in blue, the retract curve. *F*_rep_ is the repulsion force, *F*_JT_ is the jump to force, and *F*_PO_ is the pull-off force.

A schematic of the measured forces is shown in Fig. 1. The out-of-contact repulsion force (*F*_rep_) was measured in the first peak from right to left of the approaching curve, the height of the triangle that was formed in the peak from the origin to the highest part of the peak until the signal was stable in the approach curve was measured as the force. The jump to force (*F*_JT_) was measured in the minimum point of the approach curve, and the pull-off force (*F*_PO_) was measured in the minimum point of the retract curve.

#### 2.2.7 X-ray photoelectron spectroscopy analysis

The surface composition of MPT, TO, TGL and TGL-TO were investigated using X-ray photoelectron spectroscopy (XPS). The analysis was performed using a Thermo Scientific™ K-Alpha™ X-ray Photoelectron Spectrometer with a monochromatic Al K_α_ (1486 eV) X-ray source using charge compensation and a 400 μm spot size. Survey spectra were recorded with a pass energy of 200 eV and a step size of 0.4 eV, while high-resolution spectra were recorded with a pass energy of 40 eV and a step size of 0.1 eV. Three non-overlapping regions were measured for each sample. The data was processed in CasaXPS (V 2.3.16).

Peaks in the survey spectra were assigned using the Scofield library, and a linear background was used for all peaks. Deconvolution of the Ti 2p peaks obtained from the high-resolution scans was performed according to the literature^24,25^ applying two adjacent regions ranging from 451.79 eV to 461.5 eV and 461.5 to 468.0, respectively. For both regions, a Shirley background with an average width of 2 was used, and the position at 461.5 eV was offset by a factor of 18. 8 components relating to the four oxidation states of Ti (0, II, III and IV) and the doublets for the 2p_1/2_ and 2p_3/4_ states expected for each of these four peaks were fitted in all spectra. For the untreated Ti6Al4V surfaces, the positions of all peaks except the two prominent Ti(iv) and the Ti(0) 2p_3/2_ peaks were constrained according to literature values.^24,25^ The Ti(0) 2p_1/2_ peak was restrained to +6.1 eV from the Ti(0) 2p_1/2_ peak. For all other samples, only the two Ti(iv) peaks were not constrained, all other position constraints were kept constant. ESI Table 1 shows a summary of all constraints used for the treated surfaces.†

## 3 Results

### 3.1 Surface topography

**Fig. 2:**
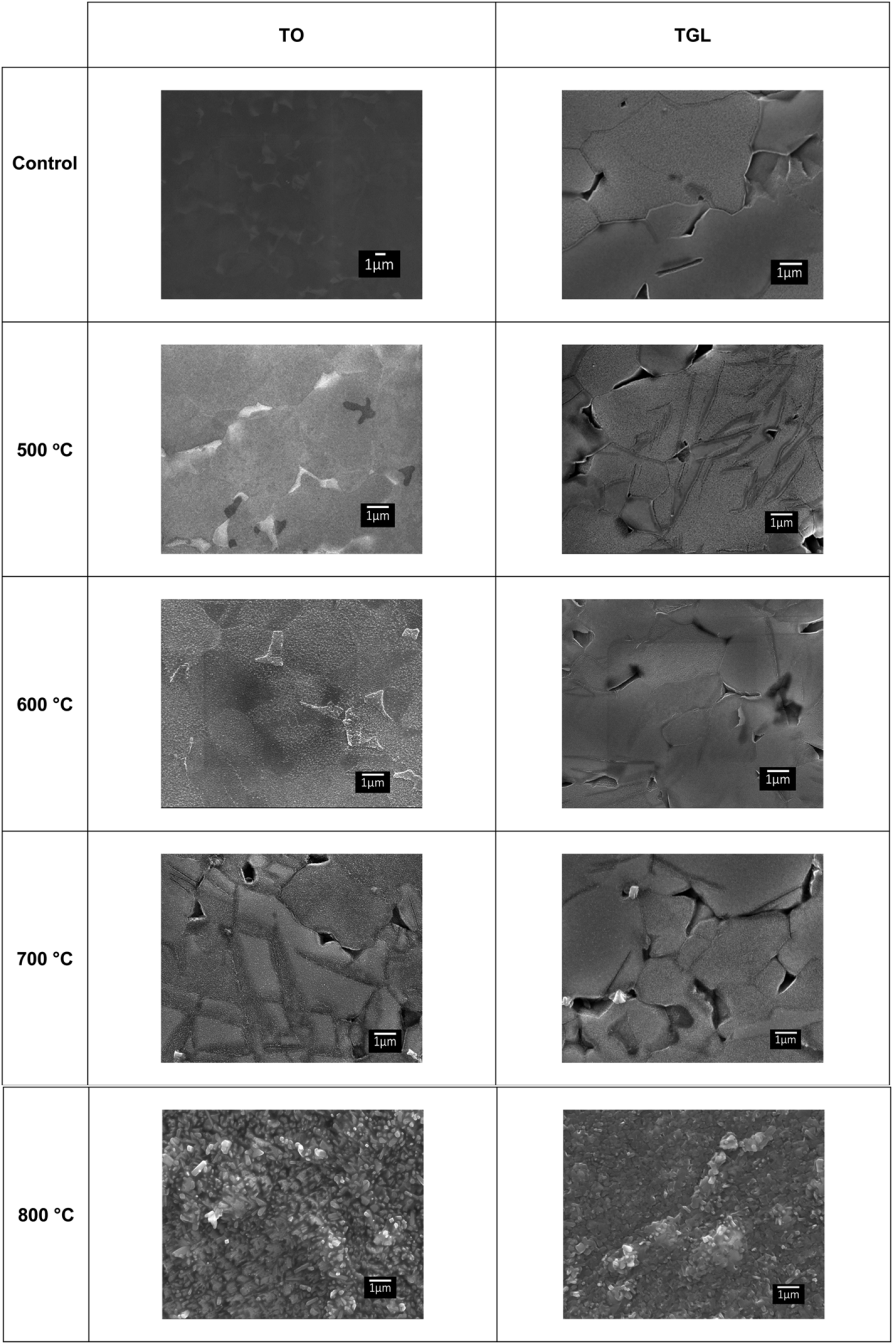
SEM micrographs of polished Ti6Al4V(MPT), thermally oxidised Ti6Al4V surfaces (TO_500°C_, TO_600°C_, TO_700°C_, TO_800°C_), H_2_O_2_/HCl chemically treated Ti6Al4V (TGL), and both chemically treated and thermally oxidised Ti6Al4V (TGL-TO_500°C_, TGL-TO_600°C_, TGL-TO_700°C_, TGL-TO_800°C_).

**Fig. 3:**
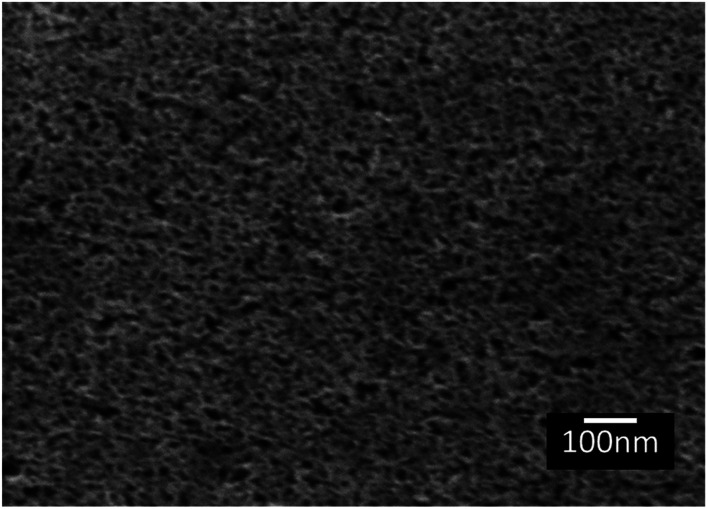
SEM micrograph of TGL Ti6Al4V modified surface.

The topography of the polished Ti6Al4V surfaces (MPT), thermally oxidised Ti6Al4V surfaces (TO_500°C_, TO_600°C_, TO_700°C_, TO_800°C_), H_2_O_2_/HCl chemically treated Ti6Al4V (TGL), and both chemically treated and thermally oxidised Ti6Al4V (TGL-TO_500°C_, TGL-TO_600°C_, TGL-TO_700°C_, TGL-TO_800°C_) are shown in Fig. 2. From the SEM micrographs, it is apparent that the surface topography increased with increasing TO temperature. The roughness values of these surfaces measured by AFM are listed in Table 2. It was observed that the surface roughness increased with increasing TO temperature. In addition, TGL-TO Ti6Al4V surfaces presented a larger roughness compared to the TGL Ti6Al4V surface. An SEM micrograph of the TGL Ti6Al4V surface showing its topography is also presented in Fig. 3. The average roughness (*S*_a_) of the modified Ti6Al4V surfaces ranged between 0.7 nm (MPT) to 121 nm (TGL-TO_800°C_).

**Table 2:** Roughness analysis of surface topography measurements for MPT, TO, TGL and TGL-TO Ti6Al4V modified surfaces by AFM shown in ESI Fig. 1. In the first column is the sample name, in the second column, the arithmetical average roughness (*S*_a_) and in the third column, the root mean square average roughness (*S*_q_). Measurements were performed using a scan window of dimensions 10 × 10 μm at a resolution of 512 × 512 pixels

Sample	AFM	
	*S*_a_ (nm)	*S*_q_ (nm)
MPT	0.7	2.1
TO_500°C_	2.3	3.1
TO_600°C_	10.2	15.2
TO_700°C_	20.6	30.9
TO_800°C_	106.9	133.5
TGL	8.5	17.0
TGL-TO_500°C_	12.0	26.7
TGL-TO_600°C_	16.8	28.2
TGL-TO_700°C_	49.0	69.2
TGL-TO_800°C_	121.0	151.4

### 3.2 Spectroscopic analysis

**Fig. 4:**
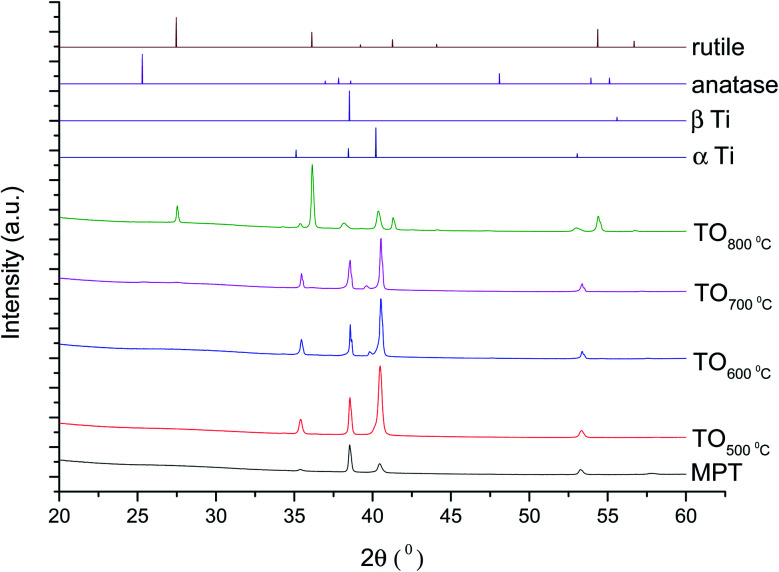
XRD diffractograms for MPT and TO Ti6Al4V modified surfaces, α-Ti (JCPDS no. 44-1294), β-Ti (JCPDS no. 44-1288), anatase (JCPDS no. 21-1272) and rutile (JCPDS no. 21-1276).

**Fig. 5:**
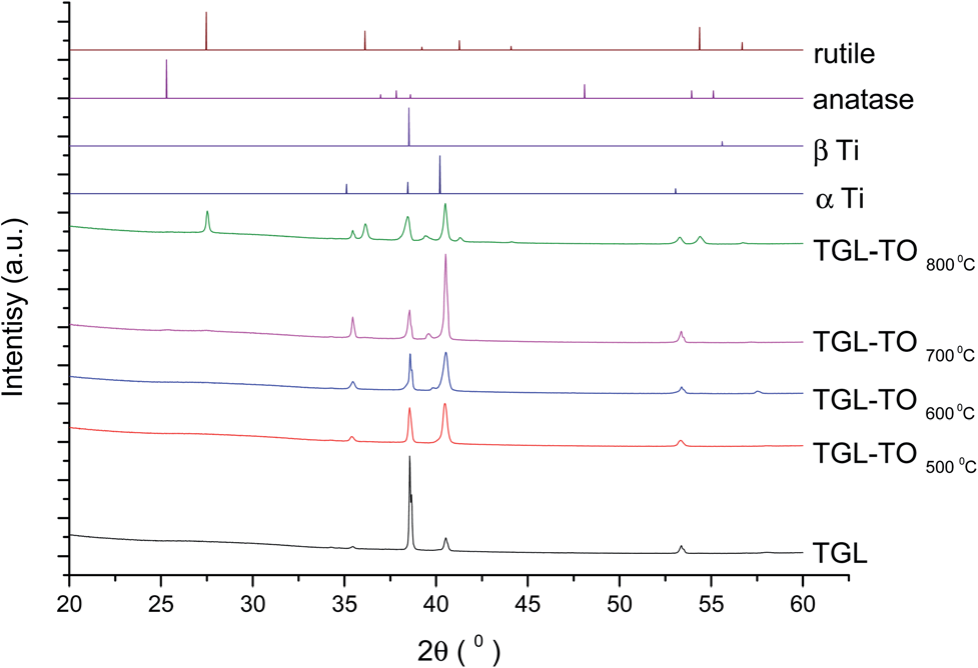
XRD diffractograms for TGL and TGL-TO Ti6Al4V modified surfaces, α Ti (JCPDS no. 44-1294), β Ti (JCPDS no. 44-1288), anatase (JCPDS no. 21-1272) and rutile (JCPDS no. 21-1276).

The XRD diffractograms of the samples with and without chemical treatment after TO with their respective controls (MPT and TGL) are shown in Fig. 4 and 5 together with the diffraction patterns of α-Ti (JCPDS no. 44-1294), β-Ti (JCPDS no. 44-1288), anatase (JCPDS no. 21-1272), and rutile (JCPDS no. 21-1276) crystal phases. The X-ray diffractograms for the samples treated at 500, 600, and 700 °C, as well as the control samples (MPT and TGL), showed the presence of α-titanium. In contrast, the XRD diffractogram of samples treated at 800 °C showed a rutile phase.

**Fig. 6:**
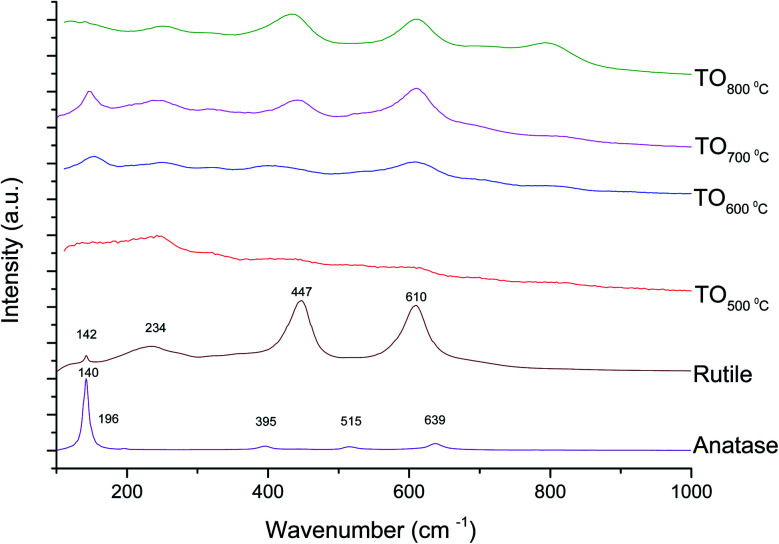
Raman spectra for anatase, rutile and TO Ti6Al4V modified surfaces.

**Fig. 7:**
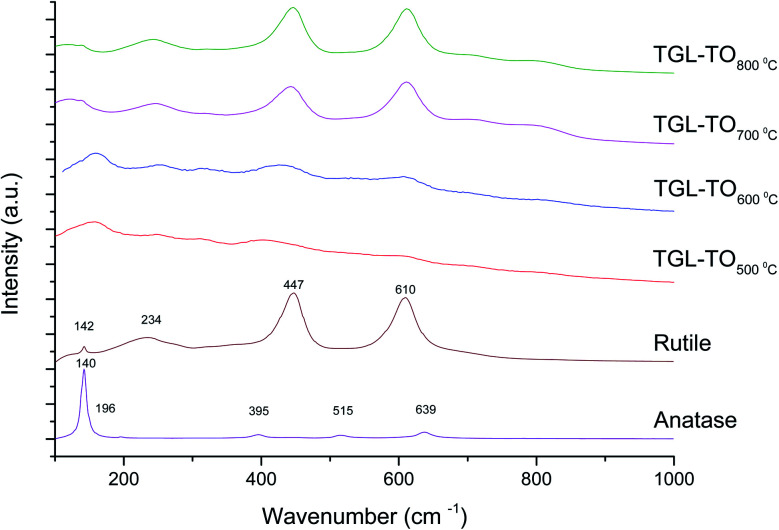
Raman spectra for anatase, rutile and TGL-TO Ti6Al4V modified surfaces.

The Raman spectra of anatase, rutile and samples with and without chemical treatment after thermal oxidation are shown in Fig. 6 and 7, respectively. Active vibrational modes of anatase in the spectrum are assigned to E_g_ at 140 cm^−1^, E_g_ at 196 cm^−1^, B_1g_ at 395 cm^−1^, A_1g_ at 515 cm^−1^, and E_g_ 639 cm^−1^.^26^ Active vibration modes of rutile are assigned to B_1g_ at 142 cm^−1^, E_g_ at 447 cm^−1^, A_1g_ at 610 cm^−1^, and E_2g_ 812 cm^−1^.^27^ The vibrational modes of Ti–O bonds were associated with the vibrational modes of anatase for the heat treatments at 500 and 600 °C and associated with rutile for the heat treatments at 700 and 800 °C for all Ti6Al4V modified surfaces.

### 3.3 Chemical composition

XPS spectra of MPT, TO, TGL, and TGL-TO Ti6Al4V modified surfaces are shown in ESI Fig. 2,† and the elemental composition obtained from these spectra is listed in Table 3. At the lower TO temperatures used, the elemental composition of the sample surface was close to that of the untreated surfaces, while higher TO temperatures altered the elemental composition notably for both the TO and TGL-TO Ti6Al4V modified surfaces. For both types of surface modification, Ti% and C% decreased when the TO temperature increased. In comparison, Al% and V% increased with increasing temperature, while the O% remained approximately constant.

**Table 3:** Composition of MPT, TO, TGL and TGL-TO Ti6Al4V modified surfaces measured by XPS

Sample	Ti 2p %	O 1s %	V 2p %	Al 2s %	C 1s %	N 1s %	aOther%
MPT	22.0 ± 0.7	52.3 ± 0.5	0.2 ± 0.0	4.2 ± 0.1	19.7 ± 0.9	0.7 ± 0.1	0.9 ± 0.1
TO_500°C_	20.4 ± 0.1	52.5 ± 0.2	0.4 ± 0.0	3.8 ± 0.1	20.0 ± 0.3	1.1 ± 0.0	1.8 ± 0.1
TO_600°C_	18.7 ± 0.8	53.3 ± 0.4	1.0 ± 0.1	4.5 ± 0.1	19.5 ± 1.0	1.1 ± 0.1	2.0 ± 0.2
TO_700°C_	15.4 ± 0.2	52.6 ± 0.4	2.4 ± 0.0	9.5 ± 0.1	18.6 ± 0.6	1.0 ± 0.1	0.5 ± 0.1
TO_800°C_	11.3 ± 0.3	51.5 ± 0.6	2.3 ± 0.0	18.9 ± 0.3	15.3 ± 1.2	0.6 ± 0.0	0.1 ± 0.0
TGL	19.2 ± 0.5	51.5 ± 0.3	0.2 ± 0.0	3.6 ± 0.1	22.3 ± 0.9	1.3 ± 0.1	1.9 ± 0.1
TGL-TO_500°C_	15.4 ± 1.9	51.1 ± 3.6	1.5 ± 0.1	9.7 ± 0.7	20.7 ± 5.6	1.3 ± 0.6	0.2 ± 0.0
TGL-TO_600°C_	13.1 ± 0.1	52.8 ± 0.8	3.0 ± 0.0	12.5 ± 0.2	17.3 ± 1.0	1.1 ± 0.1	0.1 ± 0.0
TGL-TO_700°C_	12.2 ± 0.1	52.6 ± 1.2	3.2 ± 0.1	14.4 ± 0.3	16.4 ± 1.5	1.0 ± 0.1	0.2 ± 0.1
TGL-TO_800°C_	6.5 ± 0.6	49.9 ± 1.5	2.0 ± 0.1	26.9 ± 0.2	14.0 ± 2.4	0.6 ± 0.1	0.2 ± 0.0

a Other includes Ca, P, S and Si.

**Fig. 8:**
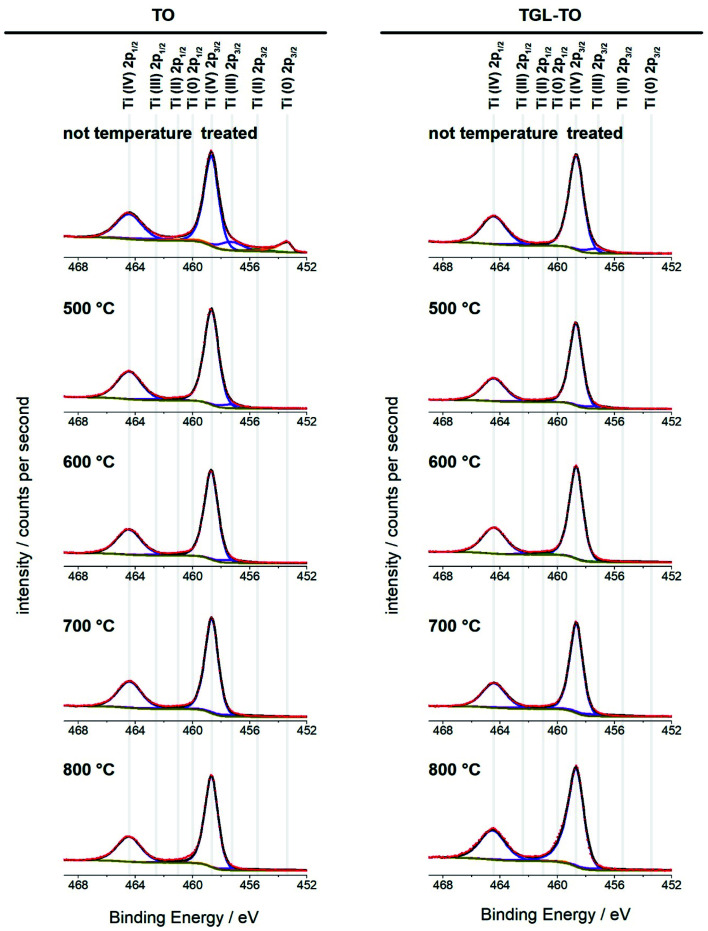
High-resolution XPS spectra of the Ti 2p peak obtained from the treated and untreated Ti6Al4V surfaces. All four oxidation states were included in the fitting for all spectra; their peak positions are indicated above the spectra. Red circles: experimental data; black line: envelope; yellow: baseline; blue: Ti(iv); purple: Ti(iii); green: Ti(ii); orange: Ti(0).

The high-resolution Ti 2p spectra of the Ti6Al4V modified surfaces (Fig. 8) are used for the curve fitting to determine the relative amounts of the four oxidation states of Ti present on the surfaces. The percentage contribution of each Ti oxidation state of the MPT Ti6Al4V modified surface is shown in Table 4. In all spectra, the predominant oxidation state was Ti(iv). Only the MPT Ti6Al4V modified surface displayed a notable second component for Ti(0). Ti(iii) was also detectable in small amounts on all samples. Ti(ii) and Ti(0) (with the exception of the MPT Ti6Al4V modified surface) were not present in any notable quantities.

**Table 4:** Relative Ti oxidation state compositions for the treated and untreated Ti6Al4V surfaces determined by curve fitting the Ti 2p peak in the XPS spectra

Sample	Ti(0) %	Ti(ii) %	Ti(iii) %	Ti(iv) %
MPT	11.1 ± 02	2.5 ± 0.1	11.8 ± 0.3	74.6 ± 0.5
TO_500°C_	0.4 ± 02	0.6 ± 0.0	5.7 ± 0.2	93.3 ± 0.1
TO_600°C_	0.4 ± 0.2	0.6 ± 0.1	5.4 ± 0.1	93.5 ± 0.2
TO_700°C_	0.4 ± 0.5	0.6 ± 0.6	5.4 ± 1.6	93.7 ± 0.9
TO_800°C_	0.1 ± 0.1	0.0 ± 0.0	9.8 ± 1.0	90.1 ± 0.9
TGL	0.7 ± 0.2	0.5 ± 0.1	6.5 ± 0.2	92.4 ± 0.3
TGL-TO_500°C_	0.0 ± 0.0	0.9 ± 0.2	5.7 ± 0.1	93.5 ± 0.2
TGL-TO_600°C_	0.0 ± 0.0	0.0 ± 0.0	8.7 ± 0.8	91.3 ± 0.8
TGL-TO_700°C_	0.1 ± 0.1	0.5 ± 0.8	7.0 ± 2.5	92.4 ± 1.6
TGL-TO_800°C_	1.4 ± 1.0	0.0 ± 0.0	7.4 ± 0.4	91.2 ± 1.0

### 3.4 Surface dynamic contact angle and force–distance curves

**Fig. 9:**
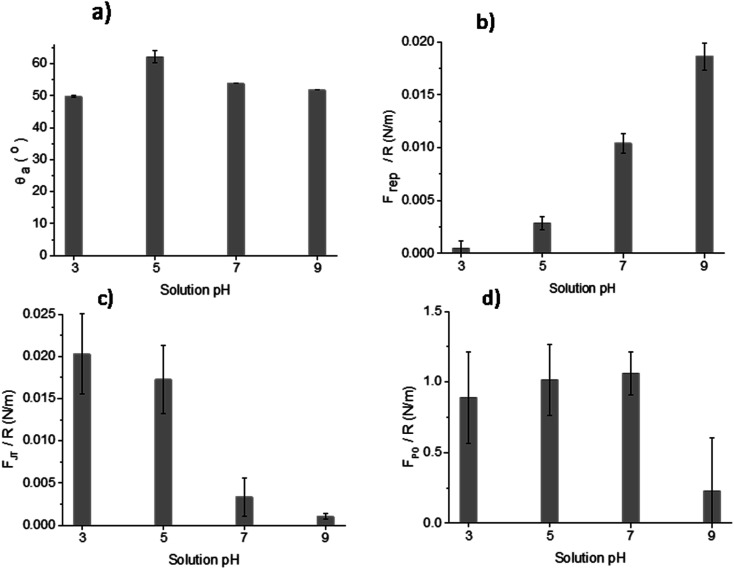
Measurements of (a) *θ*_a,_ (b) *F*_rep_, (c) *F*_JT_ and (d) *F*_PO_ force distance curves of MPT Ti6Al4V modified surface in the presence of acidic and alkaline solutions at pH 3, 5, 7 and 9.

The results of DCA and AFM adhesion measurements for the MPT surface are shown in Fig. 9. This surface presented lower roughness, *S*_a_ ∼ 0.7 nm, compared to all other treated surfaces (TGL, TO, and TGL-TO). The *θ*_a_ for MPT is shown in Fig. 9a. The *θ*_a_ was in the range of 49–53° at pH 3, 7, and 9, whereas at pH 5 the *θ*_a_ increased to 62°.

The force–distance data obtained by AFM performed against the MPT surface at pH 3, 5, 7 and 9 are shown in Fig. 9b–d. The *F*_rep_ increased with increasing pH, the *F*_JT_ was higher when pH was acidic compared to the other solutions pH, and the *F*_PO_ decreased at pH 9 compared with pH 3, 5 and 7.

**Fig. 10:**
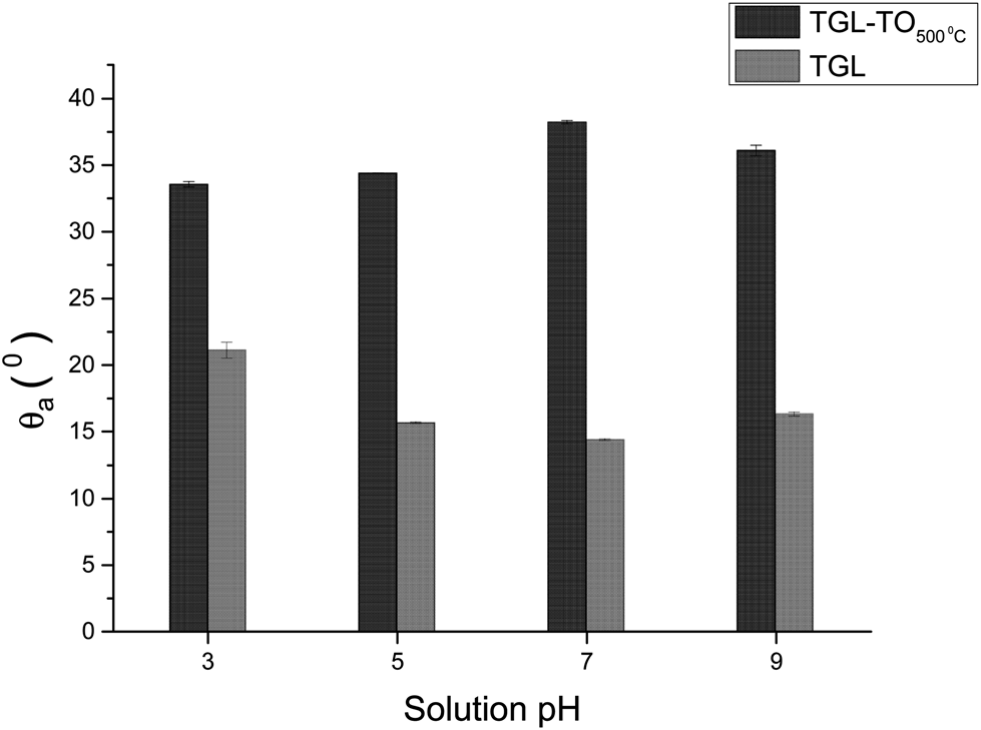
*θ*_a_ of TGL and TGL-TO_500°C_ Ti6Al4V modified surfaces at pH 3, 5, 7 and 9.

The *θ*_a_ of the TGL and TGL-TO_500°C_ surfaces are shown in Fig. 10, in which the TGL-TO_500°C_ surface exhibited a higher *θ*_a_ than the TGL surface. Furthermore, the TGL surface exhibited a lower *θ*_a_ at pH 5–9 (*θ*_a_ ∼ 15°) than at pH 3 (*θ*_a_ ∼ 20°), whereas the TGL-TO_500°C_ surface did not respond as strongly to pH change, exhibiting a minimum *θ*_a_ of 33° at pH 3, and a maximum *θ*_a_ of 38° at pH 7.

## 4 Discussion

### 4.1 Surface topography

The thermally treated Ti6Al4V surfaces exhibited a range of topographies according to the treatment temperature. During TO at temperatures above 600 °C, a crystalline TiO_2_ layer was formed, as evidenced by XRD and Raman spectroscopy analyses. It has been reported that during the TO process, oxygen penetrates the substrate.^28^ It has also been reported that aluminium diffusion,^29^ as well as oxygen diffusion,^29^ play a role in surface topography changes due to the formation of nodules,^29^ small grains^30^ and pores.^28^ In our case, the roughness of modified Ti6Al4V surfaces increased with increasing TO temperature. Table 2 shows that the roughness of the treated surfaces increased with increasing TO temperature starting from *S*_a_ ∼ 2 nm at 500 °C to *S*_a_ ∼ 100 nm at 800 °C. Similar results were obtained by Kumar *et al.*, who reported an increase in the roughness of Ti6Al4V surfaces with increasing TO temperature after treatments between 500–800 °C in air for 8–48 h.^9^ A similar trend in surface roughness with increasing temperature was also reported by Lee *et al.*^31^ for commercial purity titanium (CP-Ti) surfaces treated between 300–750 °C in air for 30 min.

On the other hand, the chemically treated surface before thermal oxidation showed pores formation, the number of which increased with increasing the temperature of the TO process. A similar effect was reported by Wang *et al.*^8^ who suggested that porous TGL was formed on the surface of chemically treated (8.8 M H_2_O_2_/0.1 M HCl aqueous solution at 80 °C for 30 min) pure titanium (CP-Ti) after TO using temperatures in the range of 300–800 °C in air.

### 4.2 Surface chemical composition

The crystal characterization of thermally oxidized samples by XRD and Raman spectroscopy are shown in Fig. 4. On the one hand, only rutile was possible to be identified by XRD on samples thermally treated at 800 °C. It was observed that α-Ti was present for all the thermally treated samples except for samples treated at 80 °C suggesting that the oxide layer formed due to TO at 800 °C was thicker than that of the samples treated at 500–700 °C. This observation about the increased oxide thickness was also reported by Kumar *et al.*^9^ who observed that the TiO_*x*_ thermally treated in air at 800 °C for 8 h consisted mainly of rutile crystals and was thicker than the TiO_*x*_ layer formed after thermal treatment at 500 °C and 650 °C for 8 h.

On the other hand, Raman spectroscopy (Fig. 6) confirmed that samples treated below 800 °C showed vibrational modes associated with both anatase and rutile crystal phases. In contrast, at 800 °C, the vibrational modes were associated only with rutile. These results are in agreement with Chang and Huang^7^ who studied the TiO_2_ crystal transformation by Raman spectroscopy. They reported that amorphous TiO_2_ and anatase were present in Ti6Al4V samples treated between 400 and 550 °C. Rutile formation began at 550 °C, whereas the metastable phase of anatase was transformed completely to rutile after heat treatment at 700 °C, suggesting that only rutile phase was formed at temperatures above 700 °C.

In contrast, the crystal characterization for chemically treated Ti6Al4V that underwent further treatment by TO at low temperatures did not show the presence of TiO_2_ crystal phases (Fig. 5). Kim *et al.*^32^ reported that the TGL obtained after treatment with NaOH and thermal treatment at 600 °C was amorphous. In our case, the broad lines at 24, 28 and 48° in the X-ray diffractogram (Fig. 5), as well as the broad Raman peaks at the vibrational modes of Ti–O bonds in the Raman spectra (Fig. 7), indicated that the oxide layer formed was indeed amorphous.

Chemical analysis of MPT surfaces confirmed the presence of Al, Ti, O, C and small amounts of V and N in the passive oxide layer. This was in accordance with the XPS study of Ti6Al4V surfaces performed by Roessler *et al.*^2^ and Milošev *et al.*^33^ who reported the presence of Al in the passive oxide as well as C, associated with adsorbed hydrocarbons. In our case, small traces of other elements (Ca, P, S and Si) were also detected on the MPT Ti6Al4V surfaces and are believed to be associated with residual elements from manufacturing not specified by the supplier.

High-resolution spectra of the Ti 2p peak showed that Ti (iv) is the main Ti oxide present (above 90%; Fig. 8 and Table 3). Some Ti (iii) was detected on all samples, whereas Ti(ii) and Ti(0) were only present in trace amounts. This indicates that the temperatures used here had little impact on the composition of the oxidation states of the surfaces. An exception of this is the untreated polished MPT surface that contained 11% Ti(0) and 11.8% Ti(iii) and therefore a slightly lower relative amount of Ti(iv) (74.6%). This result was in accordance with the study performed by Lee and Chang^34^ on Ti6Al4V native surfaces who detected the same sub-oxides measured by XPS.

The Al and V content of the oxide layer was increased with increasing the treatment temperature, as is shown in Table 3. Du *et al.*^35^ reported that Al diffused from the substrate to the outer surface as Al_2_O_3_. Al diffusion was more pronounced with increasing the treatment temperature. Kumar *et al.*^29^ came to a similar conclusion, reporting that thermally treated Ti6Al4V surfaces were Al enriched compared with untreated Ti6Al4V. The V and Al enrichment were also reported by MacDonald *et al.*^35^ in surfaces thermally treated at 600 °C. In addition, C and N were observed to be present on the surface of thermally oxidised Ti6Al4V surfaces, as shown in Table 3. Still, their levels were considerably decreased when the temperature increased above 500 °C.

The increased O/Ti ratio for the TO treated sample at 500 °C (Table 3), with and without prior chemical treatment, compared with that of the untreated sample, was mainly due to O diffusion in the substrate and consequent formation of the oxide layer. The O/Ti ratio increase at treatment temperatures above 500 °C was due to the formation of a more dense oxide layer, in agreement with Guleryuz and Cimenoglu.^28^ They reported that dense oxide layers could be obtained at temperatures between 600–800 °C.

In addition, the XPS analysis of TGL-TO Ti6Al4V surfaces, also shown in Table 3, reveals that the Al content increased with increasing the treatment temperature, whereas the change in the V was very small. Sun and Wang^36^ reported that TGL formed on the Ti6Al4V surface might act as a diffusion barrier, preventing Al and V ions diffusing to the surface from the substrate. In our case, this might be true for V; however, it was obvious that the aluminium content increased consistently with increasing the treatment temperature for the TGL-TO Ti6Al4V.

### 4.3 Contact angle and adhesion surface properties

The surface contact angle evaluated at pH 3, 5, 7 and 9 (Fig. 9a) showed variations that can be attributed to the change of the interfacial tension. These are directly related with the intermolecular forces bounded by the solid and the contacting liquid.^37^ It has been reported that the liquid surface tension of unbuffered water does not change significantly with the addition of salt concentrations up to 1 M.^38^ Consequently, changes in the contact angle titration behaviour could be attributed to the interaction of surface chemical moieties with the ionic content of the droplet through protonation and deprotonation.^38,39^

In Fig. 9a, the *θ*_a_ of the MPT surface was highest at pH 5. This maximum could be associated with the isoelectric point (IEP) of the native titanium oxide (*n*TiO_*x*_), as explained by Chau and Porter.^37^ They reported that in the absence of specific absorbed ions, the maximum angle in the contact angle titration must be related to the IEP. This was also confirmed by McCafferty and Wightman^40^ who determined the IEP of Al_2_O_3_ and Cr_2_O_3_ as the maximum value obtained by contact angle titration. According to the literature, the IEP of TiO_*x*_ occurs between pH 4–6.^41^ It has also been reported that the IEP of TiO_*x*_ is influenced by the density of hydroxyl groups present in crystal phases, contamination, and inclusion of ions.^2^ For example, the IEP of anatase can vary between pH 5.9–6.6 and of rutile between pH 4.7–6.0.^2^ Moreover, Roessler *et al.*^2^ found that the surface IEP of native oxide on Ti6Al4V was around pH 4.4. Based on the information above, the use of contact angle titration is a suitable method for accurately determining the IEP of MPT surfaces only, considering that the high roughness of thermally and/or chemically treated samples interfered in the measurement and the data obtained was more suited to a qualitative assessment of surface charge.

The AFM adhesion study of MPT surfaces reinforced the argument that the IEP is around pH 5. The AFM adhesion analysis was performed with a Si cantilever that typically has a native SiO_2_ layer with an IEP reported in some studies between pH 1 and 4 (ref. 42–44) and in some other studies at pH 3.9.^45,46^ Raiteri *et al.*^43^ reported the dependence of SiO_2_ charge as a function of pH. The lowest *F*_rep_ of MPT surface shown in Fig. 9b, and the highest attraction force shown in Fig. 9c, were observed at pH 3; this suggests that the cantilever was negatively charged and the MPT surface was positively charged. For solutions with pH > 5 it was expected that the negative charges of both SiO_2_ and the native TiO_*x*_ layer formed on the MPT surface increased in magnitude. This was verified by the calculation of *F*_rep,_ which increased with increasing pH (from pH 5) reaching the highest value at pH 9. Butt *et al.*.^47^ also reported the *F*_rep_ and attractive forces of a mica surface using an alumina probe. *F*_rep_ was only detected when the pH was above 8.1, and attractive forces occurred below pH 8.1. This was because the mica surface was negatively charged at the pH measured by Butt (between pH 3.1 and 10.4) and the alumina probe IEP was 8.1.^47^

The *F*_PO_ of the MPT surface, shown in Fig. 9a–d, exhibited their highest values at pH 5 and 7, yet a lower value at pH 9. These results can be explained considering that one of the surfaces was more negatively charged than the other, suggesting that the effect of the diffuse double-layer was involved in the interactions between the cantilever and the surface.^48^ Hence, the reduced negatively charged MPT surface (*n*TiO_*x*_) attracted water molecules than the negatively charged cantilever surface (SiO_2_). This allowed the forces in the aqueous interface to interact, generating larger adhesion forces at pH 5 and 7 compared with those at pH 9, where both surfaces were negatively charged. The reduction of the magnitude of the surface potential when the pH was closer to the IEP was reported by Walsh *et al.*^48^ They experimented on surfaces and a cantilever that were coated with TiO_2_ using atomic layer deposition (IEP at pH 5.1) and observed that the surfaces at pH 6.5 were negatively charged. Still, the charge was not as large as at pH 10, where the diffuse double-layer was large enough to create repulsion between both surfaces.

The trend of the advancing contact angle with the increase of the thermal treatment is to decrease due to roughness and topography, as evidenced in Fig. 3. However, the contact angle of TGL-TO_500°C_ surfaces was higher than that measured for TGL surfaces at all pH (Fig. 10), even when the thermally treated surface presented a higher average roughness (*S*_a_) than TGL surfaces (Table 2). This was an unexpected result. It has been reported that chemical treatment with peroxide can introduce Ti-complexes such as Ti-superoxide, TiO_2_^−^, Ti-peroxide and TiO_2_.^49–51^ However, Osaka *et al.*^52^ reported that further heat treatment in the air would result in the elimination of these Ti-complexes, leading to reduced hydrophilicity; these chemical reactions have been described by Osaka *et al.*^52^ and Tengvall and Lundström.^6^ The Ti-complexes slowly form polynuclear cations and anions leading to the precipitation of peroxotitanium hydrates (Ti(iv)O_3_(H_2_O)_*x*_ with *x* = 1, 2 or TiO_3(aq)_). The peroxotitanium hydrate species was suggested to form μ-bridges (Ti–O–Ti) and consist of Ti_2_O_5_.^6,52^ Osaka *et al.*^52^ detected the presence of –O–O– (related with the peroxotitanium hydrate species) on pure Ti treated with H_2_O_2_ samples by FT-IR at 890 cm^−1^. After the thermal treatment at 400 °C, the absence of the peak at 890 cm^−1^ in the FTIR spectra suggested that the additional thermal treatment eliminated the –O–O– groups. It is likely therefore that such an elimination of –O–O– groups might happen in the case of the TGL-TO_500°C_ surface, and for that reason, the contact angle was larger than for the TGL samples that were not thermally treated.

## 5 Conclusions

Ti6Al4V surfaces were modified by thermal oxidation and/or chemical treatment using a mixture of 90 v/v % of H_2_O_2_ 9.78 M/10 v/v % of HCl 1 M solution and were characterised by several advanced surface and structural characterisation techniques including AFM, DCA, XRD, Raman spectroscopy and XPS. The oxide layer formed on the surface exhibited different characteristics in terms of composition, topography and roughness, leading to enhanced electrostatic interactions with antimicrobial peptides. This paper presents a systematic study on the effect of thermal and chemical modifications on the quality of the oxide layer formed on a Ti6Al4V surface.

All modified Ti6Al4V surfaces showed an increase in surface roughness with increasing the thermal treatment temperature. In addition, TGL-TO samples treated at low temperatures (500–600 °C) exhibited an etched surface originated from the H_2_O_2_ treatment performed prior to the thermal treatment, whereas the TGL-TO samples treated at high temperatures presented a topography surface due to the thermal oxidation. Amorphous TiO_2_ was predominantly present in the passive TiO_*x*_ layer for all samples, but after heat treatments and with increasing the temperature, this layer became crystalline and predominantly rutile confirmed by both XRD and Raman spectroscopy.

Al and V diffusion from the substrate occurred in almost all thermally treated samples with Al content increasing with increasing the treatment temperature.

DCA analysis showed that the IEP of the MPT sample surface was around pH 5. In addition, AFM suggested that the surface was positively charged in aqueous solutions below pH 5, and negatively charged above pH 5. The *F*_PO_ values measured for the MPT surface showed, that when a strong negatively charged cantilever was used, a reduction in magnitude, as well as increased adhesion forces, were observed when the pH was close to the IEP.

Finally, the thermal treatment of TGL samples at 500 °C influenced the surface wettability. This suggested that the elimination of Ti–H_2_O_2_ during the thermal treatment at 500 °C for the latter resulted in different surface composition and topography.

## Conflicts of interest

There are no conflicts to declare.

## Supplementary Material

RA-010-C9RA11000C-s001
