# Supplementary material for: Selective modification of Ti6Al4V surfaces for biomedical applications
Source: RSC Adv. 2020 May 6;10(30):17642–52. doi: 10.1039/c9ra11000c (PMC9053614; doi:10.1039/c9ra11000c)
Supplement: RA-010-C9RA11000C-s001 [file RA-010-C9RA11000C-s001.pdf]

Electronic Supplementary Material (ESI) for RSC Advances.
This journal is © The Royal Society of Chemistry 2020

Supplementary Information

	TO	TGL
**Control**	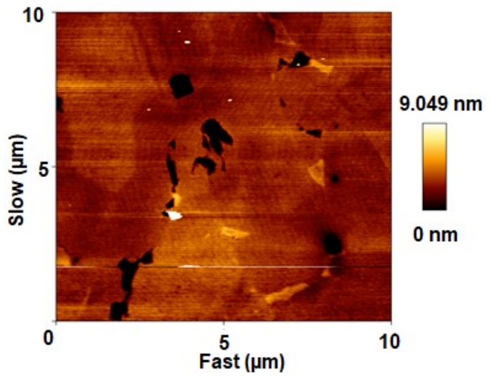	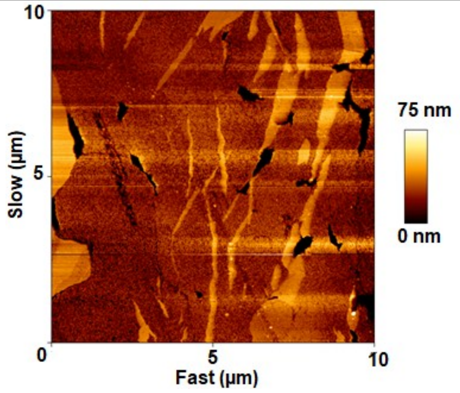
**500 °C**	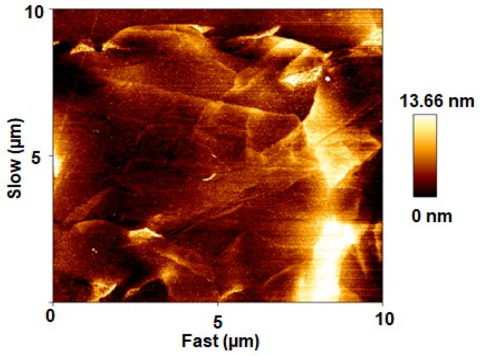	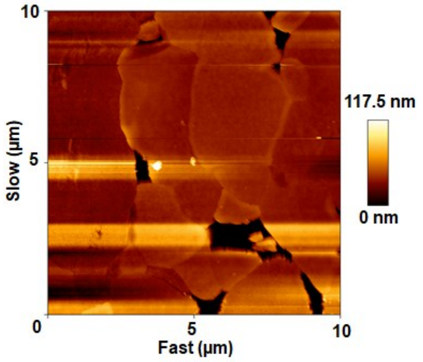
**600 °C**	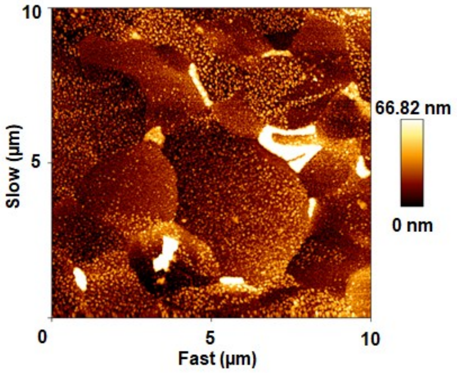	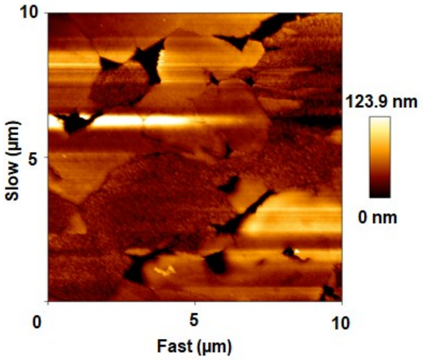

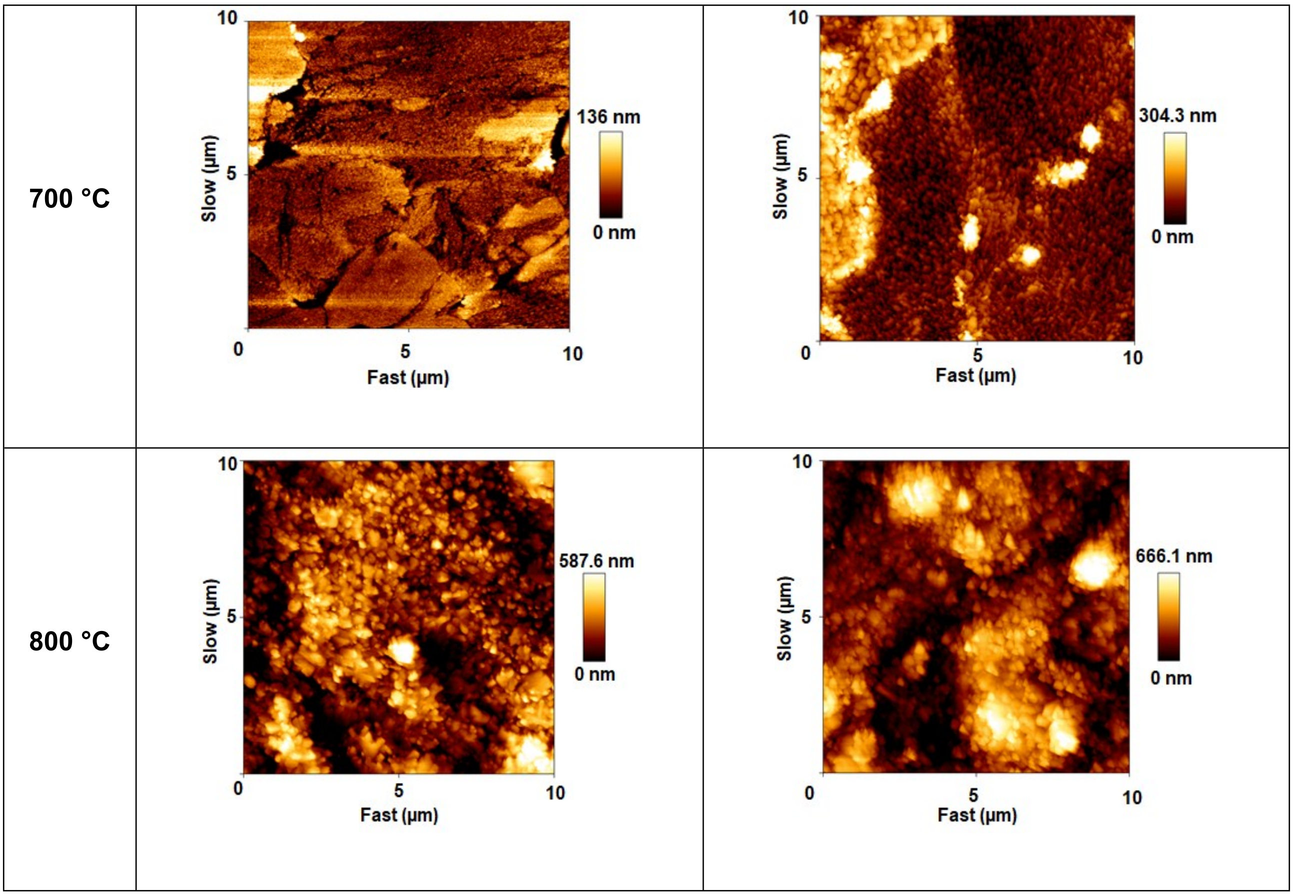

Supplementary Figure 1. AFM images 10 x 10 µm area at a resolution of 512 x 512 pixels of mirror Ti6Al4V(MPT), thermally oxidised Ti6Al4V surfaces ($TO_{500\ °C}$, $TO_{600°C}$, $TO_{700\ °C}$, $TO_{800\ °C}$), H_2O_2/HCl chemically treated Ti6Al4V (TGL), and both chemically treated and thermally oxidised Ti6Al4V (TGL-$TO_{500\ °C}$, TGL-$TO_{600\ °C}$, TGL-$TO_{700\ °C}$, TGL-$TO_{800\ °C}$).

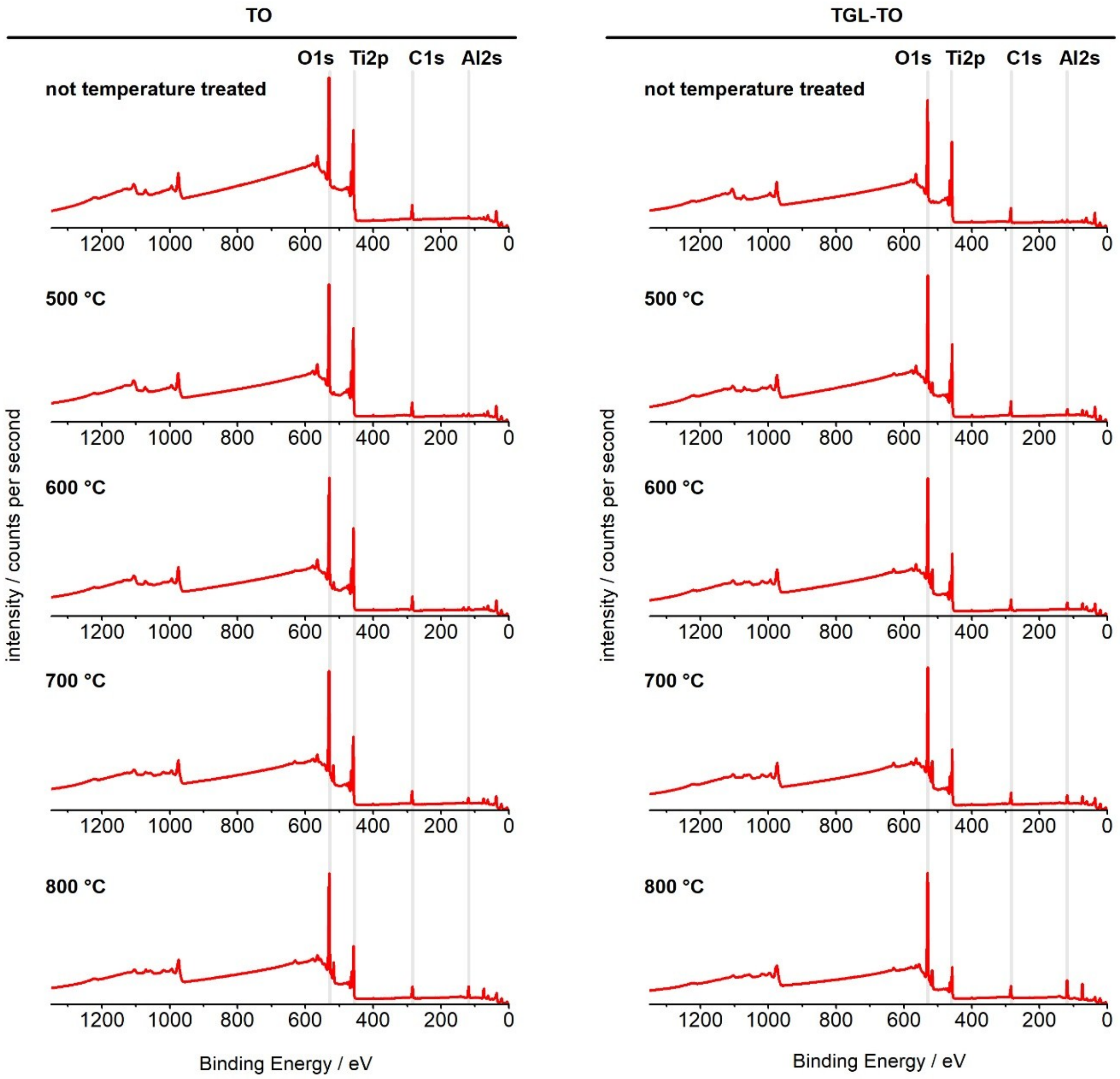

Supplementary Figure 2: XPS spectra of TO TGL-TO treated Ti6Al4V surfaces. Only the most prominent peaks are labelled for clarity.

Supplementary Table 1. Parameters used for the fitting of Ti2p high-resolution scans of treated Ti6Al4V surfaces obtained by XPS.

Component	Peak shape [a]	Area constraint	Position constraint relative to Ti(IV) 2p3/2 / eV	FWHM constraint
Ti(0) 2p1/2	LA(1.1,5,7)	0.5 x Ti(0) 2p3/2	+ 0.73	-
Ti(0) 2p3/2	LA(1.1,5,7)	-	- 5.37	2 x Ti(IV) 2p3/2
Ti(II) 2p1/2	GL(50)	0.5 x Ti(II) 2p3/2	+ 2.4	1 x Ti(III) 2p1/2
Ti(II) 2p3/2	GL(50)	-	- 3.2	1 x Ti(III) 2p3/2
Ti(III) 2p1/2	GL(50)	0.5 x Ti(III) 2p3/2	+ 3.8	-
Ti(III) 2p3/2	GL(50)	-	- 1.4	-
Ti(IV) 2p1/2	GL(50)	0.5 x Ti(IV) 2p3/2	-	-
Ti(IV) 2p3/2	A(0.3,0.1,0)GL(50)	-	-	-

[a] LA: asymmetric Lorentzian line shape; GL: Gaussian Lorentzian line shape; A()GL(): asymmetric Gaussian Lorentzian line shape
